# Complementary cooperation of an ambulance helicopter and car with medical doctors: meaning of simultaneous dispatch

**DOI:** 10.1186/cc13260

**Published:** 2014-03-17

**Authors:** K Ishii, K Kurasawa, S Tanabe, T Noguchi

**Affiliations:** 1 Oita University Hospital, Oita, Japan

## Introduction

Recently, the dispatch system with a medical doctor in pre-hospital care has made rapid progress in Japan. We usually use an ambulance helicopter or an ambulance car/rapid response car for dispatch. In this report, we analyzed the cases in which the medical doctors were dispatched using both means at the same time.

## Methods

We analyzed 29 cases with simultaneous dispatch with the medical doctors using ambulance helicopter and car during October 2012 to September 2013.

## Results

The average distance of dispatch was 25.6 km. The correspondence to the plural patients was three trauma cases. In nine cases, the ambulance car was first selected for the dispatch vehicle and the ambulance helicopter was added. In three cases, both devices were canceled on the dispatch way to the scene. In seven cases, the ambulance car was canceled. In 10 cases, the patients were transferred by the ambulance helicopter.

## Conclusion

In the selection of the dispatch means, the time to contact with the patient is the most important factor. However, the decision of the suitable means is occasionally quite difficult at the moment of first information. The ambulance helicopter and car were complementary to each other in the dispatch mean of the medical doctors.

